# TB drug susceptibility testing in high fluoroquinolone resistance settings

**DOI:** 10.5588/ijtldopen.24.0006

**Published:** 2024-05-01

**Authors:** F. Saluzzo, F. Masood, V. Batignani, F. Di Marco, U. Majeed, A. Ghazal, D.M. Cirillo, S. Tahseen

**Affiliations:** ^1^ Vita Salute San Raffaele University, Milan,; ^2^ IRCCS San Raffaele Scientific Institute, Milan, Italy;; ^3^ National TB Control Programme, Islamabad,; ^4^ National TB Reference Laboratory, Islamabad, Pakistan

**Keywords:** TB diagnosis, drug-resistant tuberculosis, Xpert^®^ MTB/XDR, TB diagnostic algorithm, FQ resistance

## Abstract

**BACKGROUND:**

The insurgence of resistance to key drugs of the BPaLM (bedaquiline + pretomanid + moxifloxacin) regimen is a major concern. In settings with widespread resistance to fluoroquinolones (FQs), like Pakistan, new technologies, such as Xpert^®^ MTB/XDR, may ensure drug resistance upfront screening. This study aims to assess MTB/XDR's performance in detecting FQs and isoniazid resistance, proposing a renewed diagnostic algorithm for drug-resistant TB (DR-TB).

**METHODS:**

This cross-sectional prospective study, approved by the local ethical committee, collected samples from people newly and previously diagnosed with TB over 6 months. Xpert^®^ MTB/RIF Ultra, MTB/XDR, Genotype^®^ MTBDR*plus*, Genotype^®^ MTBDR*sl*, culture, and phenotypic drug susceptibility testing (pDST) for relevant drugs (including bedaquiline and levofloxacin) were performed. Next-generation sequencing (NGS) resolved discordances between MTB/XDR and pDST results.

**RESULTS:**

The analysis showed that MTB/XDR has 91.5% and 88.2% sensitivity and 99.5% and 97.7% specificity in detecting respectively isoniazid (INH) and resistance to FQs, demonstrating that MTB/XDR meets the WHO targets for INH resistance detection at the peripheral level. NGS effectively resolved discordances between MTB/XDR and pDST results.

**CONCLUSIONS:**

The obtained results allowed designing the proposed diagnostic algorithm for rapid identification of DR-TB, ensuring rapid and equitable access to drug susceptibility testing for TB, ultimately improving TB care and control.

## RÉSUMÉ

**CONTEXTE:**

La recrudescence de la résistance aux médicaments clés du régime BPaLM (bédaquiline + prétomanide + moxifloxacine) est une préoccupation majeure. Dans les contextes où la résistance aux fluoroquinolones (FQ) est répandue, comme le Pakistan, de nouvelles technologies, telles que Xpert^®^ MTB/XDR, peuvent assurer un dépistage initial de la résistance aux médicaments. Cette étude vise à évaluer la performance de MTB/XDR dans la détection des FQ et de la résistance à l'isoniazide, en proposant un algorithme de diagnostic renouvelé pour la TB pharmacorésistante (DR-TB, pour l’anglais «*drug-resistant TB* » ).

**MÉTHODES:**

Cette étude prospective transversale, approuvée par le comité d'éthique local, a recueilli des échantillons de personnes nouvellement diagnostiquées et précédemment diagnostiquées avec la TB pendant 6 mois. Xpert^®^ MTB/RIF Ultra, MTB/XDR, le GenoType^®^ MTBDR*plus,* le GenoType^®^ MTBDR*sl*, la culture et des tests phénotypiques de sensibilité aux médicaments (pDST, pour l’anglais «*phenotypic drug susceptibility testing* » ) pour les médicaments pertinents (y compris la bédaquiline et la lévofloxacine) ont été effectués. Le séquençage de nouvelle génération (NGS, pour l’anglais «*next-generation sequencing* » ) a résolu les discordances entre les résultats MTB/XDR et pDST.

**RÉSULTATS:**

L'analyse a montré que le MTB/XDR a une sensibilité de 91,5% et 88,2% et une spécificité de 99,5% et 97,7% dans la détection respectivement de l'isoniazide et de la résistance aux FQ, démontrant que le MTB/XDR répond aux objectifs de l'OMS pour la détection de la résistance à l'isoniazide au niveau périphérique. NGS a efficacement résolu les discordances entre les résultats MTB/XDR et pDST.

**CONCLUSIONS:**

Les résultats obtenus ont permis de concevoir l'algorithme de diagnostic proposé pour l'identification rapide de la DR-TB, garantissant un accès rapide et équitable aux tests de sensibilité aux médicaments pour la TB, améliorant ainsi la prise en charge et le contrôle de la TB.

TB remains a major global health threat, claiming more than 1.3 million lives in 2023.^1^ In this context, the spread of *Mycobacterium tuberculosis* (MTB) strains resistant to cornerstone drugs of the TB treatment regimen, such as rifampicin (RR-TB) or both rifampicin (RIF) and isoniazid (INH) (i.e., multidrug-resistant TB, MDR-TB), has generated an epidemic within the TB silent pandemic.^2^ Currently, the regimen recommended for MDR-TB is the all-oral 6-month treatment with bedaquiline (BDQ), pretomanid, linezolid with or without moxifloxacin (BPaL(M)). This regimen has demonstrated high efficacy, lower costs and the ability to improve affected people’s lives and early recovery.^3^ In particular, the results from the TB PRACTECAL trial endorse the use of the 6-month BPaLM regimen, rather than BPaL in MDR/RR-TB patients, as this combination led to greater treatment success and less emergence of drug resistance with a similar incidence of adverse events.^4^

**Figure.**
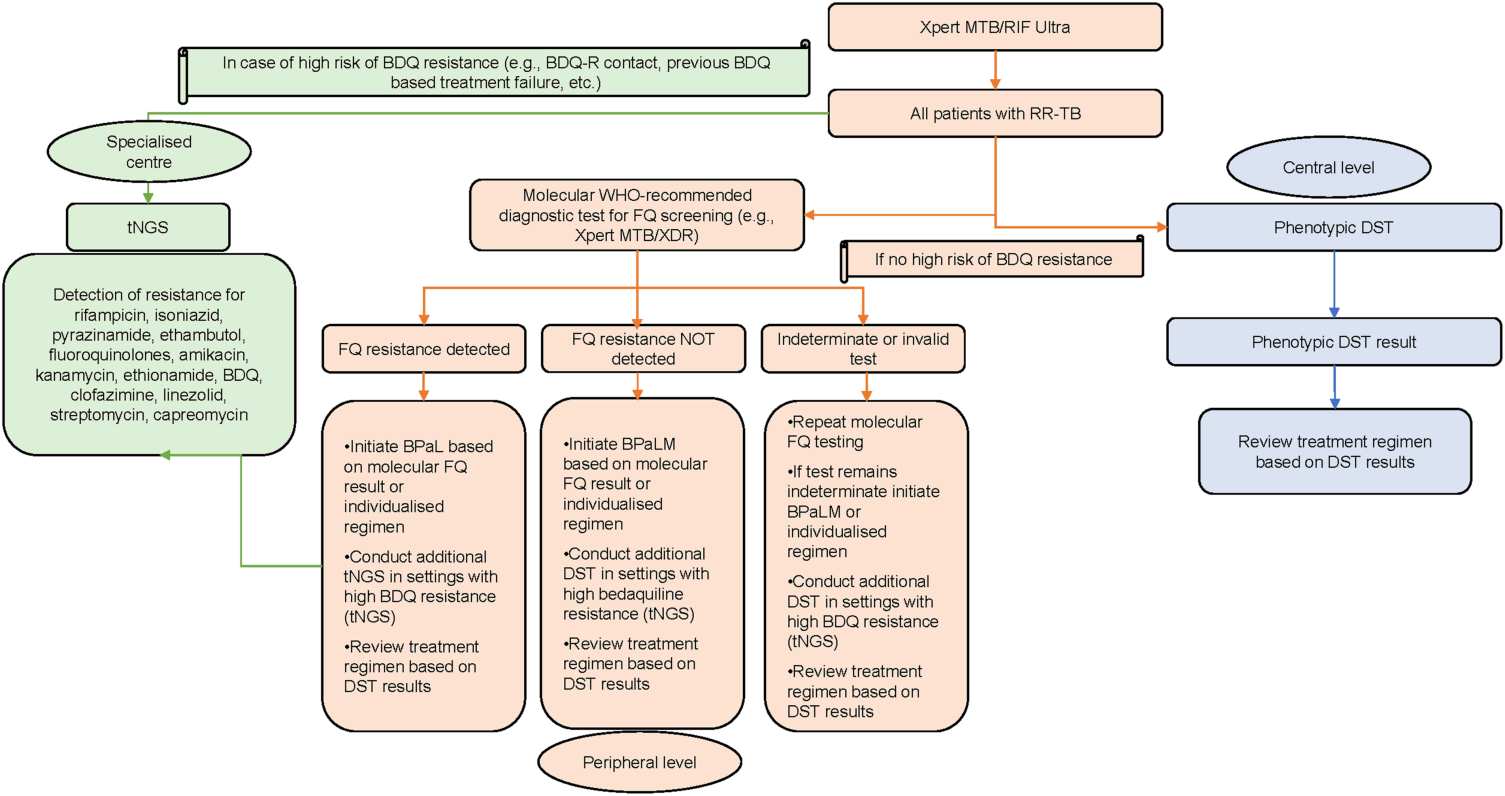
Proposed diagnostic algorithm for DR-TB molecular DST (modified from the WHO).^14^ BDQ = bedaquiline; BDQ-R = BDQ-resistant; RR-TB = rifampicin-resistant TB; tNGS = targeted next-generation sequencing; FQ = fluoroquinolone; DST = drug susceptibility testing; BPaL = BDQ + pretomanid + linezolid; BPaLM = BPaL + moxifloxacin.

Pakistan has one of the highest prevalences of drug-resistant TB (DR-TB) in the world, ranking fifth among countries with a high DR-TB burden.^5^ The prevalence of MDR-TB is currently estimated to be 2.5% among people who were newly diagnosed and 4.9% among previously treated individuals,^5^ with high fluoroquinolone (FQ) resistance in 40% of people with MDR/RR-TB.^6^

Moreover, emerging resistance to BDQ is of concern for the success of the newly recommended all-oral shorter regimen for MDR/RR-TB.^7,8^ Furthermore, INH monoresistance was reported in 9.8% of new pulmonary TB cases and high FQ resistance was associated with INH monoresistance.^6^ This is particularly relevant if we consider that FQs are main components of the WHO recommended regimen for INH-resistant TB.^9^

In this scenario, the need to improve access to testing for anti-TB drug resistance to support the rollout of shorter all-oral treatment for MDR/RR-TB and FQ-containing regimens for INH-resistant TB has clearly emerged.^10^ Even if the WHO recommends access to universal drug susceptibility testing (DST) as one of the main pillars of the 2035 End TB Strategy, the lack of access to timely DST remains a key challenge in the DR-TB cascade of care.^11,12^

The current TB diagnostic algorithm, based on the upfront testing of all presumptive TB cases with molecular WHO-recommended diagnostic tests, has favoured the detection of rifampicin resistance.^13^ A potential gamechanger strategy for early TB resistance diagnosis may be represented by the introduction of new molecular methods for the detection of resistance to key drugs, including FQ and BDQ, for the treatment of DR-TB.

The Xpert^®^ MTB/XDR (Cepheid, Sunnyvale, CA, USA), recently endorsed by the WHO, which allows the rapid detection of resistance to INH and FQs, represents a promising technique that could be implemented at a decentralised level in countries with a high DR-TB burden, such as Pakistan.^14^

This study aims to assess MTB/XDR performance in Pakistan, a high FQ resistance prevalence setting, compare it with the WHO target product profile for next-generation *Mycobacterium tuberculosis* (MTB) DST at the peripheral level, and propose an updated DR-TB diagnostic algorithm tailored to the country diagnostic network needs in the light of the newly issued WHO recommendations on the use of targeted next-generation sequencing (tNGS) for TB.^3^

## METHODS

### Study design, setting and population

In 2022, a cross-sectional prospective study was performed to estimate the performance of the index test (MTB/XDR) against the reference test, the phenotypic DST (pDST), in detecting MTB and FQs and INH resistance. For this purpose, sputum samples were obtained from 23 health centres in Pakistan, routinely linked to the National Reference Laboratory (NRL) for DST services. The samples were programmatically collected over a 6-month period from people who had been newly or previously diagnosed with TB by the Xpert^®^ MTB/RIF Ultra (Cepheid) assay, regardless of RIF resistance status.

Assuming an estimated prevalence of FQs resistance of 10% in RIF-susceptible (RS-TB) and 40% in RR-TB as well as the possibility of obtaining both pDST and MTB/XDR valid results in 80% of the recruited cohort, a sample size of a minimum of 450 individuals (300 RS-TB and 150 RR-TB) was calculated using the Fleiss formula.^15^ Data analysis was performed using R v4.3.1 (R Computing, Vienna, Austria).

### Laboratory methods

The samples were split into three aliquots. One aliquot was used for Ultra, and for samples in which MTB was detected, the second aliquot was tested using MTB/XDR, the third aliquot was processed for culture on Löwenstein-Jensen (LJ) and BACTEC™ Mycobacterial Growth Indicator Tube™ 960 (BD, Franklin Lakes, NJ, USA) liquid culture using modified Petroff’s method (N-acetyl-L-cysteine–sodium hydroxide) and the sediment was preserved for the line-probe assay (LPA). All MTB-positive cultures were identified, and pDST was performed on MTB complex isolates using BACTEC™ MGIT at critical concentrations as per WHO recommendations.^16–18^ The following drugs were tested: RIF, INH, streptomycin, ethambutol, pyrazinamide, amikacin, levofloxacin, BDQ, clofazimine, linezolid and delamanid.

LPA was performed using Genotype^®^ MTBDR*plus* (Hain Lifescience, Nehren, Germany), and Genotype^®^ MTBDR*sl* (Hain Lifescience) on the sputum sediment. Samples with sufficient volume for being split into only two aliquots were analysed directly with LPA without performing Ultra testing. Whole-genome sequencing (WGS) was performed on samples with discordant results between MTB/XDR and the pDST. tNGS was performed on specific strains that had low coverage at WGS. Both tNGS and WGS were performed on inactivated cultures shipped from Pakistan NRL to the Emerging Bacterial Pathogens Unit, Supranational Reference Laboratory, IRCCS San Raffaele Scientific Institute, Milan, Italy.

WGS was performed on Illumina NextSeq 500 (Illumina, San Diego, CA, USA) and MiniSeq platforms with paired-end Nextera XT library preparation following the manufacturer’s instructions. Sequencing data were aligned to the reference genome (GenBank number NC_000962.3) with the MTBseq pipeline.^19^ tNGS was performed with the DeeplexMyc-TB kit version (GenoScreen, Lille, France), as previously described,^20^ and sequences were then analysed using the DeeplexMyc-TB Web Application 1.0.3. Samples quality was assessed using FastQC (Babraham Bioinformatics, Babraham, UK). Number of polymerase chain reaction (PCR) cycles during library preparation were 15 for WGS and 35 for tNGS. All sequences have been uploaded on NCBI (bio project number PRJNA1083925).

Sequencing results were interpreted using the updated version of the WHO catalogue of MTB complex mutations associated with drug resistance that is currently in press.^21^ The study workflow and variables are reported in the supplementary material in Supplementary Figure S1.

### Ethical considerations

The study was approved by the Institutional Review Board Ethics Committee of the Common Management Unit for TB, AIDS and Malaria, Ministry of National Health Services Regulations & Coordination, Islamabad, Pakistan (F. No. IRB-CMU-2021-15). Informed consent was obtained from each participant. For the purposes of this study, all information that could lead to patient identification was excluded.

## RESULTS

### Description of the study population

In 2022, a total of 612 sputum samples were programmatically referred to the NRL for DST services. At the testing of samples at NRL, MTB was confirmed in 85.8% (*n* = 525) of the specimens, 501 using Xpert^®^ MTB/RIF (Cepheid), and 24 by LPA. The RIF resistance status was defined based on Ultra or LPA results at the NRL. Among 525 samples evaluated, 192 were RIF-resistant (RR-TB), and 333 were RS-TB. Further details on the participants and specimen characteristics are provided in the Supplementary Data.

### Performance of Xpert MTB/XDR in detecting MTB

Among 525 samples tested by MTB/XDR, MTB was detected in 506 (96.4%, 95% confidence interval [CI] 94.3–97.7). MTB/XDR was able to detect MTB positivity in 100% of the samples classified as high and medium with Ultra and in more than 99% of low samples (Table 1). Compared with the standard for MTB detection (MGIT™ culture), MTB/XDR provided a positive result in 506/525 MTB/XDR with an error rate of 0.19%, whereas for MGIT™ culture, 45/525 (8.6%) cultures were contaminated and 121/525 (23.0%) did not grow MTB. To further explore the possible reasons for the lack of MTB growth, the culture recovery was analysed in relation to Ultra semiquantitative results and history of previous TB treatment (Supplementary Table S2).

**Table 1.** Comparison of the performance of the Xpert MTB/XDR assay results and culture/pDST in the diagnosis of TB and drug resistance against the MTB semiquantitative results.

Xpert MTB/RIF Ultra-MTB semi quantitative results*	​	MTB/XDR assay results			Culture and pDST results	
		MTB detected	INH: interpretable results	FQ: interpretable results	Positive (MTBC)	DST results
		*n* (%)	*n* (%)	*n* (%)	*n* (%)	*n* (%)
Total	525	506 (96)	499 (95)	495 (94)	359 (68)	333 (63)
High MTB	218	218 (100)	218 (100)	218 (100)	203 (93)	189 (87)
Medium MTB	89	89 (100)	88 (99)	89 (100)	72 (81)	64 (72)
Low MTB	126	125 (99)	123 (98)	123 (98)	55 (44)	52 (41)
Very low MTB	59	47 (80)	45 (76)	39 (66)	6 (10)	5 (8)
MTB traces	9	4 (50)	2 (22)	3 (33)	3 (33)	3 (33)
NA	24	23 (96)	23 (96)	23 (96)	20 (83)	20 (83)

* Based on the cycle threshold.

pDST = phenotypic drug susceptibility testing; MTB = *M. tuberculosis*; INH = isoniazid; FQ = fluoroquinolone; MTBC = MTB complex; NA = not available.

### Performance of Xpert MTB/XDR in detecting isoniazid resistance

Of the 333 samples for which pDST was available for INH, two were excluded because, one resulted invalid result and in the second, an error occurred at the MTB/XDR. The total error rate of the MTB/XDR test was 0.9%. As shown in Table 2, the sensitivity of the test was 91.5% (95% CI 85.4–95.7), and the specificity was 99.5% (95% CI 97.3–99.9) regardless of RIF resistance. The predictive positive value (PPV) was 99.2% (95% CI 95.4–99.9), and the negative predictive value (NPV) was 94.8% (95% CI 90.8–97.7). In the RR-TB cohort, the assay proved to be highly sensitive and specific (sensitivity 91.9%; specificity 95.8%). However, in the RS-TB cohort, the assay was highly specific (100%) but less sensitive (89.5%).

**Table 2.** Analysis of the accuracy of Xpert MTB/XDR in detecting resistance to isoniazid regardless of rifampicin results and in RR-TB and RS-TB cohorts.

Parameters	All	RR-TB	RS-TB
	(*n* = 330)	(*n* = 135)	(*n* = 195)
	% (95% CI)	% (95% CI)	% (95% CI)
Sensitivity	91.5 (85.4–95.7)	91.9 (85.2–96.2)	89.5 (66.9–98.7)
Specificity	99.5 (97.3–99.9)	95.8 (78.9–99.9)	100.0 (97.9–100)
PPV	99.2 (95.4–99.9)	99.0 (94.7–99.9)	100.0 (80.5–100)
NPV	94.8 (90.8–97.7)	71.9 (53.3–86.3)	98.9 (96.0–99.9)

RR-TB = rifampicin-resistant TB; RS-TB = rifampicin-susceptible TB; CI = confidence interval; PPV = positive predictive value; NPV = negative predictive value.

### Performance of Xpert MTB/XDR in detecting fluoroquinolones resistance

Regarding FQ resistance, 332/525 samples had a pDST available for FQs and a valid MTB/XDR result. The MTB/XDR error rate in this cohort was 0.3%. The accuracy of the analysis, regardless of RIF resistance (Table 3), was as follows: sensitivity, 88.2% (95% CI 78.7–94.4); specificity, 97.7% (95% CI 94.8–99.1). The PPV was 91,8% (95% CI 88.2–94.4), and the NPV was 96,5% (95% CI 93.8–98.1). For FQs, the test had greater sensitivity (91.1%, 95% CI 78.8–97.5) in the RR-TB cohort and higher specificity in the RS-TB cohort (98.8%, 95% CI 95.7–99.9). A comparison of the INH and FQ results obtained with the MTB/XDR, LPA and pDST is provided in Supplementary Tables S3, S4A and S4B.

**Table 3.** Analysis of the accuracy of the test in detecting resistance to FQs in RR-TB and RS-TB cohorts.

Parameters	All	RR-TB	RS-TB
	(*n* = 330)	(*n* = 135)	(*n* = 195)
	% (95% CI)	% (95% CI)	% (95% CI)
Sensitivity	88.2 (78.7–94.4)	91.1 (78.8–97.5)	83.9 (66.3–94.6)
Specificity	97.7 (94.8–99.1)	95.7 (89.2–98.8)	98.8 (95.7–99.9)
PPV	91.8 (83.0–96.9)	91.1 (78.8–97.5)	92.9 (76.5–99.1)
NPV	96.5 (93.5–98.4)	95.7 (89.2–98.8)	97.0 (93.2–99.0)

RR-TB = rifampicin-resistant TB; RS-TB = rifampicin-susceptible TB; CI = confidence interval; PPV = positive predictive value; NPV = negative predictive value.

### Comparison of Xpert® MTB/XDR with WHO Target Product Profile for DST at the peripheral level

The obtained accuracy results were compared with the minimum and optimal requirements established by the WHO target product profile (TPP) for DST at peripheral centres.^22^ Detailed comparison results are displayed in Supplementary Table S5. The diagnostic sensitivity of MTB/XDR for MTB detection compared with that of MGIT™ culture completely fulfils the optimal requirement defined by the WHO. Moreover, the sensitivity and specificity of MTB/XDR for detecting INH resistance reached the established minimal requirements when calculated irrespective of RIF resistance status. Regarding FQ resistance, MTB/XDR fulfils the minimal sensitivity requirements in the RR-TB cohort, whereas the specificity of the test (96%) does not reach the minimal WHO requirement. However, in the RS-TB cohort, the specificity criteria were met (98.8%, 95% CI 91.8–100), but MTB/XDR did not reach the minimal target sensitivity.

Furthermore, to ascertain if the scope, pricing, and operational characteristics of MTB/XDR, in addition to performance, satisfy the relevant WHO TPP, a comparison with the relevant data was performed and the results are summarised in Supplementary Table S6. In summary, MTB/XDR satisfied most of the WHO TPP requirements, resulting an almost point-of-care test, which can be used by healthcare workers with limited training. The pricing of the modular platform is in line with the WHO parameters (US$3,860 per single module), but the price per cartridge is slightly elevated (US$19.8 per cartridge being the WHO minimal requirement of US$15 per cartridge for the detection of at least RIF, INH and FQ). Operationally, the test satisfies all the other minimal characteristics required by the WHO, including sample type and volume, biosafety measures, and time to result.

### Sequencing of MTB strains with discordant results between the pDST and Xpert MTB/XDR

WGS was performed on 28 samples with discordant results between pDST and MTB/XDR for INH (*n* = 12) or FQs (*n* = 16). On 7 low-quality samples (6 were INH-resistant using pDST and susceptible on MTB/XDR and 1 was susceptible using pDST and resistant to the MTB/XDR), tNGS was performed. Detailed results of the obtained results and their comparison with the newly published WHO catalogue for mutations^23^ are reported in the Supplementary Data and in Supplementary Tables S7A and S7B. In summary, the rate of very major errors of MTB/XDR for INH resistance detection was 1.1% and for FQs was 0.6%, well below the 1.5% US Food and Drug Administration’s limit.^24^

### A new algorithm for DR-TB diagnosis

Considering the results of the performance analysis against the WHO TPP, Pakistan MDR-TB, pre-extensively drug-resistant TB (pre-XDR-TB) and XDR-TB epidemiology and the sequencing results, a possible update of the algorithm that allows a rapid molecular approach to target both FQs and BDQ resistance is proposed below. In summary, in the case of a high risk of BDQ resistance, the sample will be immediately shipped to the NRL where tNGS will be implemented. All other samples will be screened on site for FQs and INH resistance using MTB/XDR. DR-TB treatment will be started according to the MTB/XDR results and modified, if needed, once sequencing or pDST results are available for the other drugs included in the regimen (e.g., BDQ). Considering the expected number of samples, the transport timeline and the test “hands on” and analysis time, this approach foresees reducing the current turnaround time (TAT) of the result of molecular WHO-recommended rapid diagnostic tests (mWRDs) from 10 days (LPA TAT) to 1 day and the TAT of confirmatory diagnosis of resistance from 30 days (pDST TAT) to 2 weeks (Figure).

## DISCUSSION

Decentralisation of the DST is a pivotal step in achieving of universal TB DST.^25^ In high TB burden settings with extreme DR-TB prevalence, such as Pakistan, FQs and INH are among the priority anti-TB compounds to be tested at the decentralised level.^22^ MTB/XDR and LPA are currently the main tools available for the rapid diagnosis of pre-XDR-TB.^26^ Nonetheless, LPA needs trained personnel and dedicated infrastructures to be performed, limiting possible implementation at the peripheral level.^14^

In this study, the accuracy analysis demonstrated that MTB/XDR met the WHO targets for implementation at the peripheral level for INH DST irrespective of the RIF result.^22^ Moreover, the test performed well in detecting MTB even if its positivity rate decreased in the ‘very low’ and ‘traces’ samples. This is expected because of the lower limit of detection of MTB/XDR (136 colony forming units (CFU)/mL of unprocessed sputum) compared to that of Ultra (15.6 CFU/mL of unprocessed sputum).^14,27^

Regarding FQ resistance, MTB/XDR reached the WHO sensitivity target in only the RR-TB cohort but not the required specificity. In the RS-TB cohort, the test met the specificity requirements but not the sensitivity requirements. Beyond performance, the MTB/XDR scope is in line with WHO requirements, as well as the costs of the platform/instrument and the needed infrastructures, training, and maintenance. However, the cost of each MTB/XDR cartridge is greater than that recommended by the WHO, especially since the same MTB/XDR cartridge cannot be used to detect RIF, INH and FQs. This factor needs to be considered, as it may affect the future sustainability of the test.

Nevertheless, the integration of MTB/XDR in the diagnostic algorithm may provide numerous benefits to the overall TAT between diagnosis and initiation of appropriate treatment and may beneficially support a diagnostic network overburden by the high sample number that needs to be transported and analysed every year. Considering the reduced performance of the pDST in this context, the suboptimal sample transportation system and the limited specificity of the MTB/XDR, the need for a complementary molecular test to support the FQ DST at the central level has clearly emerged. The recently released WHO rapid communication on the use of tNGS for the diagnosis of DR-TB supports the use of this method for TB resistance detection. If capacity is built, the implementation of tNGS at the central level in high-burden countries may also support the rapid detection of BDQ resistance. In Pakistan, emerging resistance to BDQ is a major concern for the success of the newly recommended all-oral shorter regimen for MDR/RR-TB.^9,28^ Therefore, the combined use of these two tools, MTB/XDR at the peripheral level and tNGS at the central level, may support the early identification of resistance to key drugs, including BDQ, administered in the new oral regimen and provide several operational advantages. According to recent publications, tNGS, as mWRDs, may contribute to easing the burden on central laboratories related to the execution of phenotypic DST and other tests, such as LPAs, ultimately improving the TAT of resistance diagnostics.^29,30^

In our analysis, tNGS was demonstrated to be a reliable tool for the identification of FQ resistance. Moreover, tNGS has already proven to be, in similar settings, a reliable tool to provide results in a timely manner, allowing the modification of TB treatment accordingly.^31,32^ Therefore, the tNGS test may effectively overcome the discussed constraints of the pDST and support pre-XDR-TB and XDR-TB diagnosis in Pakistan.

The main limitation of this study is the lack of a cost-benefit analysis comparing the costs of the implementation of the renewed algorithm with the estimated benefits. Nevertheless, it is well known that countries with a high TB burden could earn significantly more than they spend on TB diagnosis and treatment if they invest in TB care and control in an organised and consistent way.^33^

In conclusion, this renewed approach may be applied not only in Pakistan but also in several other high-burden countries where high FQs resistance and emerging BDQ resistance are detected. This strategy can ultimately contribute to the achievement of universal DST in these settings and ensure equitable access to MDR and pre-XDR-TB diagnosis, improving effectiveness and efficacy of the diagnostic network.

## Supplementary Material


