# Supplementary material for: TB drug susceptibility testing in high fluoroquinolone resistance settings
Source: IJTLD Open. 2024 May 1;1(5):230–5. doi: 10.5588/ijtldopen.24.0006 (PMC11249602; doi:10.5588/ijtldopen.24.0006)
Supplement: Supplementary file 1 [file iutld_ijtld_open_24.0006_supplementarydata1.pdf]

http://dx.doi.org/10.5588/ijtldopen.24.0006

SUPPLEMENTARY DATA

TB drug susceptibility testing in high fluoroquinolone resistance settings. Novel solutions for an evolving challenge

Study workflow

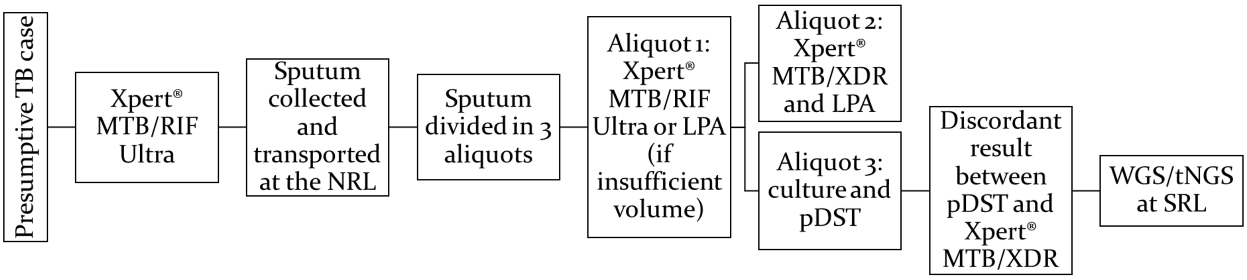

Figure S1 Samples and strains workflow. Abbreviations: NRL: national reference laboratory, SRL: supranational reference laboratory, LPA: Line probe assay

Variables and data sources

To define the general performance of the test in detecting MTB, the results obtained with Xpert® MTB/XDR (MTB detected, MTB not detected, indeterminate, invalid and error) were compared with the semiquantitative results obtained with Xpert® MTB/RIF Ultra (high, medium, low, very low and traces).
To establish Xpert® MTB/XDR performance against the reference test (pDST MGIT™ 960) in detecting MTB and FQs and isoniazid (INH) resistance sensitivity, specificity, positive predictive value (PPV) and negative predictive value (NPV) were calculated.
To ascertain if the performance, scope, pricing and operational characteristics of Xpert® MTB/XDR satisfy the relevant WHO TPP, a comparison with the relevant data was performed.

Demographic data

Table S1 Demographic and clinical characteristics of participants in the study.

Variables (gender and age)	Total No=192		Total No =333		Total No =525	
**Gender**	**No**	%	**No**	%	**No**	%
Female	94	49.0%	145	43.5%	239	45.5%
Male	98	51.0%	188	56.5%	286	54.5%

Age Group (Yrs)	No	%	No	%	No	%
<5	0	0.0%	1	0.3%	1	0.2%
5-14	6	3.1%	2	0.6%	8	1.5%
15-19	25	13.0%	29	8.7%	54	10.3%
20-24	24	12.5%	43	12.9%	67	12.8%
25-34	37	19.3%	62	18.6%	99	18.9%
35-44	26	13.5%	43	12.9%	69	13.1%
45-54	32	16.7%	64	19.2%	96	18.3%
55-64	28	14.6%	40	12.0%	68	13.0%
>65	10	5.2%	43	12.9%	53	10.1%
NA	4	2.1%	6	1.8%	10	1.9%
Minor (<18yrs)	25	13.0%	17	5.1%	42	8.0%
**History of ATT**	**No**	%	**No**	%	**No**	%
NO	65	33.9%	80	24.0%	145	27.6%
NA	40	20.8%	129	38.7%	169	32.2%
Yes	87	45.3%	124	37.2%	211	40.2%
**Province**	**No**	%	**No**	%	**No**	%
Punjab	183	95.3%	302	90.7%	485	92.4%
ICT	9	4.7%	8	2.4%	17	3.2%
AJK	0	0.0%	22	6.6%	22	4.2%
KPK	0	0.0%	1	0.3%	1	0.2%

Acronyms: FL-ATT: first-line treatment; SL-ATT: second-line treatment; NA: information not available; F: female; M: male; yo: years old.

Among the included participants, 54,5% were males and majority of the participants were aged between 18 and 55 years old. The participants included 42 minors (<18 years old), accounting for 8% of the study population. Among the enrolled participants, 40.2% had a history of previous TB treatment.

Specimens' characteristics

Among RR specimens, 184/192 had a valid result for INH and 185/192 for FQs by Xpert® MTB/XDR. The results of both pDST (the standard test for drug susceptibility testing) and Xpert MTB/XDR were available for INH for 135/192 samples and for FQs for 137/192 samples.

According to the results of the pDST, 82.8% (95% CI, 75.4-88.8) of the RR-TB cohort participants were resistant to INH (MDR-TB), and 32.8% (95% CI, 25.5-41.1) were resistant to FQs (preXDR-TB). Among RS specimens, 315/333 had interpretable results for INH and 310/333 for FQs by Xpert® MTB/XDR.

The results for both pDST and Xpert® MTB/XDR were available for INH and FQs for 195/333 samples. Among the RS-TB cohort, 9.7% (95% CI, 6.3-14.7) of specimens were resistant to INH, and 15.9% (95% CI, 11.4-21.7) were resistant to FQs. The results of the pDST and of Xpert® MTB/XDR are reported in the flowchart below.

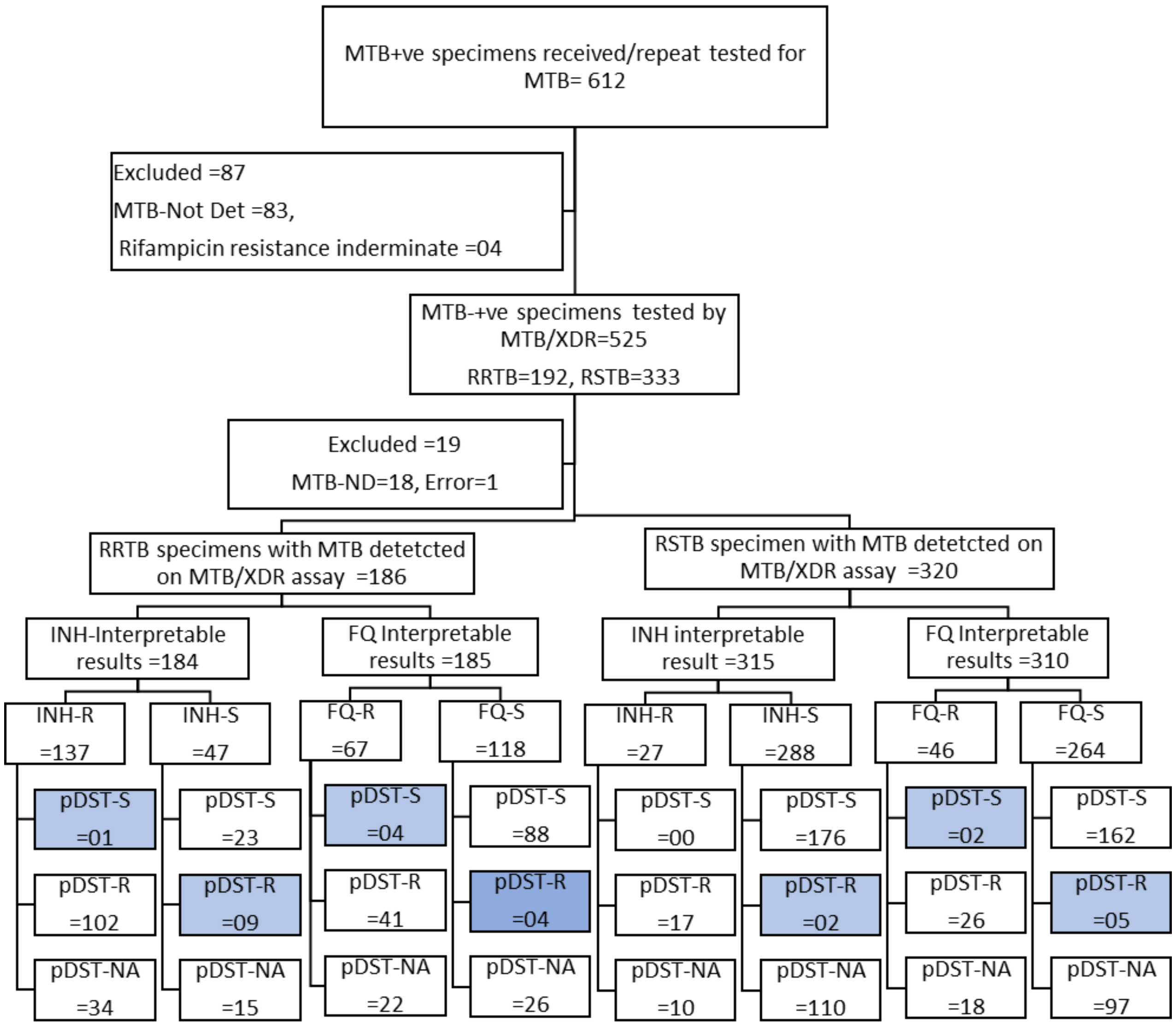

Figure S2 Flowchart of samples Xpert® MTB/XDR and pDST results

Supplementary results

Table S2 Effect of previous TB treatment and bacillary load on culture recovery in specimens from rifampicin-resistant and -sensitive patients.

	**History of previous TB treatment**								
**Ultra semi MTB quantitative and culture**	**All specimens**			**Rifampicin Resistant**			**Rifampicin sensitive**		
	**All**	**NO/NA**	**Yes**	**All**	**No/Na**	**Yes**	**All**	**NO/NA**	**Yes**
**All**	**525**	**314**	**211**	**192**	**105**	**87**	**333**	**209**	**124**
Culture +	359	239	120	155	91	64	204	148	56
%	68.4%	76%	56.9%	80.7%	86.7%	73.6%	61.3%	70.8%	45.2%
**p Value**			**0.00**			**0.022**			**0.00**
**MTB-H**	**218**	**139**	**79**	**112**	**59**	**53**	**106**	**80**	**26**

Culture +	203	133	70	99	54	45	104	79	25
%	93%	96%	89%	88%	92%	85%	98%	99%	96%
**p Value**			**0.044**			**0.243**			**0.172**
**MTB-M**	**89**	**51**	**38**	**29**	**16**	**13**	**60**	**35**	**25**
Culture +	72	46	26	26	16	10	46	30	16
%	81%	90%	68%	90%	100%	77%	77%	86%	64%
**p Value**			**0.009**			**0.043**			**0.046**
**MTB-L**	**126**	**72**	**54**	**26**	**16**	**10**	**100**	**56**	**44**
Culture +	55	37	18	20	14	6	35	23	12
%	44%	51%	33%	77%	88%	60%	35%	41%	27%
**p Value**			**0.044**			**0.098**			**0.145**
**MTB-VL**	**59**	**30**	**29**	**10**	**4**	**6**	**49**	**26**	**23**
Culture +	6	5	1	1	1		5	4	1
%	10%	17%	3%	10%	25%	0%	10%	15%	4%
**p Value**			**0.075**			**0.197**			**0.197**
**MTB-T**	**9**	**5**	**4**	**6**	**4**	**2**	**3**	**1**	**2**
Culture +	3	3	0	2	2	0	1	1	0
%	33%	60%	0%	33%	50%	0%	33%	100%	0%
**p Value**			**0.058**			**0.221**			
**NA***	**24**	**17**	**7**	**9**	**6**	**3**	**15**	**11**	**4**
Culture +	20	15	5	7	4	3	13	11	2
%	83%	88%	71%	78%	67%	100%	87%	100%	50%
**p Value**			**0.313**			**0.259**			**0.011**

Acronyms: MTB det: MTB detected; MTB-ND: MTB not detected; NA: not available

According to the performed analyses, culture recovery appeared to be affected in paucibacillary specimens and in samples collected from participants with a history of previous TB treatment. In summary, 15/121 (12,39%) not grown samples were categorized as high and medium with Xpert® MTB/RIF Ultra, 57/121 (47,1%) were reported as low and 46/121 (38,01%) as very low/traces.

Table S3: Comparison of isoniazid and fluoroquinolone results of MTB/XDR assay, Line probe assay and phenotypic DST results.

Drug	gDST results			pDST	
	MTB/XDR	LPA	Number results	R	S
INH	R	R	112	112	0
INH	R	S	7	6	1
INH	R	NA	1	1	0
INH	S	S	204	11	193
INH	S	R	4	0	4
INH	S	NA	2	0	2

INH	NA*	S	3	1	2
FQ	R	R	59	57	2
FQ	R	S	7	5	2
FQ	R	NA	7	5	2
FQ	S	S	242	7	235
FQ	S	R	3	1	2
FQ	S	NA	14	1	13
FQ	NA*	S	1	0	1

LPA performance for INH: sensitivity =86.2%. specificity: 98%, Positive predictive value: 96.6%, NPV 91.6%, LPA performance for FQ: sensitivity 82.9%, Specificity - 98.3%, PPV -93.5 and NPV 95.2%. Acronyms: gDST – Genotypic drug susceptibility testing; pDST- Phenotypic drug susceptibility testing; INH-Isoniazid; LPA-Line probe assay; FQ-Fluroquinolone: R-Resistant, S-Sensitive, NA-Not available

Table S4A Performance of first line LPA in detecting resistance to isoniazid in rifampicin resistant and rifampicin sensitive specimens

LPA performance characteristics in isoniazid resistance detection	ALL		RRTB		RSTB	
	n=330		n=136		n=194	
	%	95%CI	%	95%CI	%	95%CI
Sensitivity	86.2%	79.0-91.6	87.4%	79.7-92.9	78.9%	54.4-93.9
Specificity	98.0%	95.0-99.5	84.0%	63.9-95.5	100.0%	97.9-100
PPV	96.6%	91.4-99.1	96.0%	90.2-98.9	100.0%	78.1-100
NPV	91.6%	87.0-94.9	60.0%	42.1-76.1	97.8%	94.1-99.4

Table S4B Performance of second line LPA in detecting resistance to fluoroquinolones in rifampicin resistant and rifampicin sensitive specimens

LPA performance characteristics in FQ resistance detection	ALL		RRTB		RSTB	
	n=312		n=124		n=188	
	%	95%CI	%	95%CI	%	95%CI
Sensitivity	82.9%	72.0-90.8	90.0%	76.3-97.2	73.3%	54.1-87.7
Specificity	98.3%	95.8-99.5	96.4%	89.9-99.3	99.4%	96.5-99.9
PPV	93.5%	84.3-98.2	92.3%	79.1-98.4	95.7%	78.1-99.9
NPV	95.2%	91.8-97.5	95.3%	88.4-98.7	95.2%	90.7-97.9

Table S5 Comparison between WHO target product profile for DST at peripheral level and Xpert® MTB/XDR accuracy results obtained in the study. Modified from World Health Organization, 2021[1]

	WHO TPP Characteristic	Xpert® MTB/XDR result

Performance parameter	Minimal requirements	Optimal requirements	Overall	RRTB cohorts	RSTB cohorts
**Diagnostic sensitivity for TB detection**	>80% for a single test when compared with 2 liquid cultures (MGIT)	>95% for a single test when compared with 2 liquid cultures (MGIT)	99,7% (95%CI 98,2-100) compared with MGIT culture	-	-
**Diagnostic sensitivity for DST compared with phenotypic DST as a reference standard**	>90% for detection of phenotypic resistance	>95% for detection of phenotypic resistance	**INH:** 91,5% (95% CI: 85.4-95.7)	**INH:** 91.9% (95% CI 85.2-96.2)	**INH**: 89.5% (95% CI: 66.9-98.7)
			**FQs:** 88,2% (95% CI 78.7-94.4)	**FQs**: 91.1% (95%CI 78.8-97.5)	**FQs:** 83.9% (95% CI 66.3-94.6)
**Diagnostic specificity for DST compared with phenotypic DST as a reference standard**	≥98% for any anti-TB drug for which the test can identify resistance when compared with the phenotypic reference standard		INH: 99.5% (95% CI 97.3-99.9)	**INH:** 95.8% (95% CI 78.9-99.9)	**INH:** 100% (95%CI 97.9-100)
			**FQs:**97,7% (95%CI 94.8-99.1)	**FQs**: 95,7% (95%CI 89.2-98.8)	**FQs:** 98,8% (95%CI 95.7-99.9)
**Indeterminate results during DST**	<10%	<3%	**INH:** 0,6%	-	-
			**FQs**: 0%		
**Multiuse Platform**	Yes (achievable)	Yes (demonstrated)	Demonstrated multiuse platform		

Table S6 Comparison between WHO target product profile for DST at peripheral level and Xpert® MTB/XDR characteristics summarized in WHO guidelines for TB diagnosis. Modified from World Health Organization, 2021[1]

Characteristic	Minimal requirements	Optimal requirements	Xpert® MTB/XDR
**Scope**			
**Goal**	Diagnosis of TB disease and detection of drug resistance to provide rapid triage of patients and identification of adequate treatment regimen (first-line treatment versus second line	Diagnosis of TB disease and detection of drug resistance to inform decision-making about the optimal (individualized)regimen	Rapid triage (less than 90 mins) of drug resistance for Isoniazid and FQs allowing identification of the adequate treatment regimen

	treatment)		
**Priority of anti-TB agents for testing**	Rifampicin, Isoniazid, FQs and BDQ	In order of decreasing importance all minimal +<br>1.Pyrazinamide, Linezolid, Pretomanid/Delamanid, Clofazimine<br>2.Amikacin<br>3.Any additional drug in WHO guidelines	Detection of FQs and Isoniazid
**Target population**	People of all ages in need of evaluation for TB and those requiring drug resistance assessment		No age constraints, allows both detection of MTB and resistance to FQs and isoniazid
**Target user of the test**	Healthcare worker with minimal or moderate training	Healthcare worker with minimal training	Healthcare worker with moderate training
**Setting**	Peripheral Level	Point of Care	Classified as almost point of care test
**Pricing**			
**Price of individual test**	RIF+INH+FQs<br>15 US$	RIF+INH+FQs<br>5 US$	INH+FQs<br>19,8 US$
**Capital cost for the instrument**	Less than 20.000 US$	Less than 5.000 US$	10-colour GeneXpert® modules range from US$ 3.860 (single module kit) to US$ 72.350
**Operational**			
**Sample type**	Sputum and other clinically relevant specimens for TB	Unprocessed sputum and other clinically relevant specimens for TB and other diseases	Unprocessed sputum and other clinically relevant specimens for TB
**Sample volume**	0,5-2 mL	0,1-10 mL	Sputum sediment 0,5-2,5 mL<br>Unprocessed sputum 1- 4 mL
**Time to result**	<6 hours	<30 minutes	<90 minutes
**Biosafety**	Requirements are similar to those for smear microscopy (low risk TB laboratory)		Low risk TB laboratory

**Maintenance**	Preventive maintenance is not needed more than once a year	Preventive maintenance is not needed more than once every 2 years	Preventive maintenance is not needed more than once a year

Table S7A Results of WGS and tNGS analysis for samples with isoniazid discordant results between Xpert® MTB/XDR, pDST and Genotype® MTBDRplus.

**Sample #**	**RR/ RS**	**MTB semiquantitative results**	**INH Results/Interpretation**			**WGS result and interpretation according to WHO catalogue**	**tNGS result and interpretation according to WHO catalogue**
			**pDST**	**Xpert® MTB/ XDR**	**Genotype® MTBDRplus**		
X- 297	RR	MTB-H	S	R	WT	**Not determined**	Negative per MTBC or low coverage
X-048 XDR048 -D	RR	MTB-H	R	S	WT	**Not determined**	Variant katG_indels detected. **Associated with resistance**
X-086	RR	MTB-H	R	S	WT	**Not determined**	**No drug resistance associated** variants detected in any gene target.
X-145	RR	MTB-L	R	S	WT	**Not determined**	**No drug resistance associated** variants detected in any gene target.
X-227	RR	MTB-H	R	S	WT	Variant katG_Q127P detected. **Uncertain significance**	
X-345 D-0109-22	RR	MTB-H	R	S	WT	Variant katG_S140N detected.	-

						**Uncertain significance**	
X-378	RR	MTB-M	R	S	WT	**Not determined**	**No drug resistance associated** variants detected in any gene target.
X-486	RR	MTB-H	R	S	WT	Variant katG_D142G detected. **Uncertain significance**	-
X-487	RR	MTB-H	R	S	WT	**Not determined**	Negative per MTBC or low coverage
X-627	RR	MTB-H	R	S	WT	Variant katG_D94A detected. **Uncertain significance**	-
X-308	RS	MTB-H	R	S	WT	**Not determined**	Variant inhA_S94A detected. **Uncertain significance**
X_672	RS	MTB-H	R	S	WT	**No drug resistance associated** variants detected in any gene target.	-

Acronyms: RRTB: rifampicin-resistant TB, RSTB: rifampicin TB; R: Resistant; S: sensitive; WT: wild type; MTB-H: MTB high; MTB-M: MTB medium; MTB-L: MTB low.

Among twelve INH discordant results between Xpert® MTB/XDR and pDST, one sample was INH resistant and eleven were INH sensitive according to Xpert® MTB/XDR (Table 5A). For 2/12 samples (one INH resistant and one INH sensitive according to Xpert® MTB/XDR), even if both WGS and tNGS were performed, it was not possible to obtain a valid result due to the low quality of the specimens. Of the remaining ten discordant samples, in 4/10 (X-86, X-145, X-378 and X-672), no drug resistance-associated variants were detected in any gene target of the analyses performed, confirming the Xpert® MTB/XDR results. In 5/10 samples (X-227, X-345, X-486, X-627, X-308), variants classified as of "uncertain significance" in the WHO catalogue were identified. In the last case (X-048), tNGS confirmed resistance because of the presence of a frameshift mutation, which was not detectable by Xpert® MTB/XDR.

Table S7B Results of WGS and tNGS analysis for samples with fluoroquinolone discordant results between Xpert® MTB/XDR, pDST andGenotype® MTBDRplus.

Sample #	Rif status	MTB semiquantitative results	FQ results/Interpretation			WGS result and interpretation according to WHO catalogue [20]
			pDST	Xpert® MTB/XDR	Genotype® MTBDRsl	
X-060<br>D-2274-21	RR	MTB-H	R	S	WT	Variants gyrA_G668D, gyrA_S95T, gyrA_E21Q detected<br>**Not associated with resistance**
X-226<br>D-0053-22	RR	MTB-H	R	S	WT	Variant gyrA_D94A detected<br>**Associated with resistance**
X-344<br>D-0311-22	RR	MTB-M	R	S	R-Ind	Variant gyrA_A288D detected<br>**Uncertain significance**
X-489<br>D-0376-22	RR	MTB-M	R	S	R	Variant gyrA_A456V detected<br>**Uncertain significance**
X-115<br>D-2377-21	RR	MTB-H	S	R<br>gyrB2-mut	R-Inf	Variant gyrB_E501D detected<br>**Associated with resistance**
X-197<br>D-0110-22	RR	MTB-L	S	R<br>gyrA1-mutB, gyrA2-mutA, gyrA3-mutB	NA	Variant gyrA_A90V detected<br>**Associated with resistance**
X-228<br>D-0063-22	RR	MTB-H	S	R<br>gyrA2-mutA, gyrA3-mutB, gyrB2-mut	WT	Variant gyrB_N499T detected<br>**Uncertain significance**
X-462<br>D-0439-22	RR	MTB-T	S	R<br>gyrA1-mutB, gyrA2-mutA, gyrA3-mutB	R-Ind	Variant gyrB_T500A detected<br>**Uncertain significance**
X-359<br>D-0172-22	RS	MTB-H	R	S	WT	Variants gyrA_E21Q, gyrA_S95T, gyrA_G668D detected<br>**Not associated with resistance**

X-360 D-0173-22	RS	MTB-H	R	S	WT	Variants gyrA_E21Q, gyrA_S95T, gyrA_G668D detected **Not associated with resistance**
X-577 D-0908-22	RS	MTB-H	R	S	WT	Variants gyrA_E21Q, gyrA_S95T, gyrA_G668D detected **Not associated with resistance**
X-612 D-1160-22	RS	MTB-M	R	S	WT	Variants gyrA_E21Q, gyrA_S95T, gyrA_G668D detected **Not associated with resistance**
X-615 D-1161-22	RS	MTB-M	R	S	WT	Variants gyrA_S95T, gyrA_E21Q, gyrA_G668D detected **Not associated with resistance**
X-510 D-0565-22	RS	MTB-L	S	R gyrA3-mutB	R	Variant gyrA_D94G detected **Associated with resistance**
X-642 D-1213-22	RS	MTB-L	S	R gyrA1-mutC	WT	Variants gyrA_S95T, gyrA_E21Q, gyrA_G668D detecetd **Not associated with resistance**

Acronyms: RRTB: rifampicin-resistant TB; RSTB: rifampicin TB; R: Resistant; S: sensitive; WT: wild type; R-Ind: resistance indeterminate; R-Inf: Resistance inferred; MTB-H: MTB high; MTB-M: MTB medium; MTB-L: MTB low.

The 15 strains with discordant FQ results (six resistant to FQs and ten susceptible by Xpert® MTB/XDR) were analysed with WGS. Of 6 samples resistant to FQs by Xpert® MTB/XDR, one (X-642) had two resistant variants detected in gyrA that were not associated with resistance, consequently confirming the pDST result. In three strains reported to be FQs resistant by Xpert® MTB/XDR, variants associated with FQs resistance were identified by WGS, disproving the pDST result. In the remaining 2 samples, variants of uncertain significance were detected.
Among the 10 discordant results reported as sensitive to FQs by Xpert® MTB/XDR, in one sample, a well-known variant (gyrA_D94G) conferring resistance to FQs was detected by WGS, confirming the pDST result. Nonetheless, LPA performed on the same aliquot of Xpert® MTB/XDR at the arrival of the sample at NRL confirmed that this strain was wild type for FQs. Therefore, the possibility of preanalytical error cannot be excluded. In seven samples, resistant variants were detected, but none were associated with resistance, disproving the result of the pDST. In two samples, variants of uncertain significance were detected, not reported in the Xpert® MTB/XDR panel of variants. All results are reported in Table 7B.

Bibliography

1. World Health Organization. *Target product profile for next-generation drug-susceptibility testing at peripheral centres*. Geneva, https://www.who.int/publications/i/item/9789240032361 (2021, accessed 18 July 2023).
